# Recent Advances in Information Technology

**DOI:** 10.1155/2014/746479

**Published:** 2014-06-19

**Authors:** Fei Yu, Chin-Chen Chang, Yiqin Lu, Jian Shu, Yan Gao, Guangxue Yue, Zuo Chen

**Affiliations:** ^1^Peoples' Friendship University of Russia, Moscow 117198, Russia; ^2^Feng Chia University, Taichung 40724, Taiwan; ^3^South China University of Technology, Guangzhou 510006, China; ^4^Nanchang Hangkong University, Nanchang 330063, China; ^5^Henan Polytechnic University, Jiaozuo 454000, China; ^6^Jiaxing University, Jiaxing 314001, China; ^7^Hunan University, Changsha 410082, China

The topic of recent advances in information technology has attracted a wide range of articles on technology theory, applications from many aspects, and design methods of information technology. Reviewing the papers in this topic, it is clear that all fields such as computer science, cloud computing, wireless sensor network, prediction, image annotation, and storage have been involved. And the publications about recent advances in information technology tackled significant recent developments in the fields mentioned above, both of a foundational and applicable character.

Also, we can easily find that most contributors regard “information technology” as synonymous with tools such as the computer, mobile, and tablet and such issues as instructional design, mobile learning, social networking, and open sources. Through the topic's development, research designs are appropriate for studying the potential of information technology applications under controlled situations.

This special issue includes a collection of 100 papers selected from 466 submissions to 36 countries or districts. All submitted papers followed the same standard (peer-reviewed by at least three independent reviewers) as applied to regular submissions.

The paper entitled “*A self-adaptive parameter optimization algorithm in a real-time parallel image processing system*” by G. Li et al. proposed an adaptive load capacity balance strategy on the servo parameters optimization algorithm (ALBPO) to improve the computing precision and achieve high detection ratio while not reducing the servo circle.

The paper entitled “*Improved algorithm for gradient vector flow based active contour model using global and local information*” by J. Zhao et al. proposes an improved approach based on existing gradient vector flow methods. Main contributions of this paper are a new algorithm to determine the false part of active contour with higher accuracy from the global force of gradient vector flow and a new algorithm to update the external force field together with the local information of magneto static force.

In the paper entitled “*A topology visualization early warning distribution algorithm for large-scale network security incidents*” by H. He et al. a comprehensive early warning system is presented in this paper, which combines active measurement and anomaly detection. The key visualization algorithm and technology of the system are mainly discussed.

The paper entitled “*A novel approach to word sense disambiguation based on topical and semantic association*” by X. Wang et al. proposes a novel approach to word sense disambiguation based on topical and semantic association. For a given document, supposing that its topic category is accurately discriminated, the correct sense of the ambiguous term is identified through the corresponding topic and semantic contexts.

In the paper entitled “*A fast map merging algorithm in the field of multirobot SLAM*” by Y. Liu et al. a map merging algorithm based on virtual robot motion is proposed for multirobot SLAM. The thinning algorithm is used to construct the skeleton of the grid map's empty area, and a mobile robot is simulated in one map.

The paper entitled “*Hexahedral localization (HL): a three-dimensional hexahedron localization based on mobile beacons*” by L. Liu et al. proposes a three-dimensional range-free localization scheme named hexahedral localization. In the scheme, the space is divided into a lot of hexahedrons. Then, all the unknown nodes are located by utilizing the perpendicular properties of the trajectory.

The paper entitled “*A two-level cache for distributed information retrieval in search engines*” by W. Zhang et al. proposes a distribution strategy of the cache data. The experiments prove that the hit rate, the efficiency, and the time consumption of the two-level cache have advantages compared with other structures of cache.

This paper by Z. Wang “*Analysis of DNS cache effects on query distribution*” studies the DNS cache effects that occur on query distribution at the CN top-level domain (TLD) server, and the approximate TTL distribution for domain name is inferred quantificationally.

The paper entitled “*The generalization error bound for the multiclass analytical center classifier”* by Z. Fanzi and M. Xiaolong presents the multiclass classifier based on analytical center of feasible space (MACM). This multiclass classifier is formulated as quadratic constrained linear optimization and does not need repeatedly constructing classifiers to separate a single class from all the others.

The paper entitled “*The estimate for approximation error of neural network with two weights*” by F. Zeng and Y. Tang constructs a new BP neural network and proves that the network could approximate any nonlinear continuous function.

In the paper entitled “*An automatic image inpainting algorithm based on FCM*” by J. Liu et al. an automatic image inpainting algorithm which automatically identifies the repaired area by fuzzy C-mean (FCM) algorithm is proposed. FCM algorithm classifies the image pixels into a number of categories according to the similarity principle, making the similar pixels cluster into the same category as possible.

The paper entitled “*Using the high-level based program interface to facilitate the large scale scientific computing*” by Y. Shang et al. makes further research on facilitating the large-scale scientific computing on the grid and the desktop grid platform. The related issues include the programming method, the overhead of the high-level program interface based middleware, and the anticipated data migration.

The paper by N. Feng and C. Zheng entitled “*A cooperative model for IS security risk management in distributed environment”* develops a cooperative model for IS security risk management in a distributed environment. In the proposed model, the exchange of security information among the interconnected IS under distributed environment is supported by Bayesian networks (BNs).

The paper entitled “*Vocal emotion of humanoid robots: a study from brain mechanism*” by Y. Wang et al. explored the brain mechanism of vocal emotion by studying previous researches and developed an experiment to observe the brain response by fMRI, to analyze vocal emotion of human beings.

The paper entitled “*Real-time tracking by double templates matching based on timed motion history image with HSV Feature*” by Z. Li et al. presents a novel method with appearance model described by double templates based on timed motion history image with HSV color histogram feature (tMHI-HSV).

The paper entitled “*An improved piecewise linear chaotic map based image encryption algorithm*” by Y. Hu et al. proposes a symmetric cryptographic system using MPWLCM chaotic system to encrypt grayscale image. The scheme possesses high sensitivity to plain image and key, so it has a good ability to resist differential attack.

The paper entitled “*A Sarsa(*λ*)-based control model for real-time traffic light coordination*” by X. Zhou et al. proposes Sarsa(*λ*)-based real-time traffic control optimization model that can maintain the traffic signal timing policy more effectively. The Sarsa(*λ*)-based model gains traffic cost of the vehicle, which considers delay time, the number of waiting vehicles, and the integrated saturation from its experiences to learn and determine the optimal actions.

J. Wu et al. in their paper entitled “*Mixed pattern matching-based traffic abnormal behavior recognition*” propose a trajectory pattern learning method based on dynamic time warping (DTW) and spectral clustering. The paper introduced the DTW distance to measure the distances between vehicle trajectories and determined the number of clusters automatically by a spectral clustering algorithm based on the distance matrix.

In the paper entitled “*A reward optimization method based on action subrewards in hierarchical reinforcement learning*” by Y. Fu et al. a hierarchical reinforcement learning method based on action subrewards is proposed to solve the problem of “curse of dimensionality,” which means that the states space will grow exponentially in the number of features and low convergence speed.

In the paper entitled “*Constructing better classifier ensemble based on weighted accuracy and diversity measure*” by X. Zeng a weighted accuracy and diversity (WAD) method is presented, a novel measure used to evaluate the quality of the classifier ensemble, assisting in the ensemble selection task.

The paper entitled “*Simplified process model discovery based on role-oriented genetic mining*” by W. Zhao et al. proposes a genetic programming approach to mine the simplified process model. Using a new metric of process complexity in terms of roles as the fitness function, we can find simpler process models.

The paper entitled “*An improved topology-potential-based community detection algorithm for complex network*” by Z. Wang et al. puts forward a mass calculation method for complex network nodes, which is inspired from the idea of PageRank algorithm.

In “*Gait correlation analysis based human identification*” by J. Chen, silhouette correlation analysis based human identification approach is proposed. By background subtracting algorithm, the moving silhouette figure can be extracted from the walking images sequence.

The paper entitled “*Information spread of emergency events: path searching on social networks*” by W. Dai et al. collects Internet data based on data acquisition and topic detection technology, analyzes the process of information spread on social networks, describes the diffusions and impacts of that information from the perspective of random graph, and finally seeks the key paths through an improved IBF algorithm.

The paper entitled “*Application of butterfly Clos-network in network-on-chip*” by H. Liu et al. studied the topology of network-on-chip (NoC). By combining the characteristics of the Clos-network and butterfly network, a new topology named BFC (butterfly Clos-network) network was proposed.

The paper entitled “*A linear method to derive 3D projective invariants from 4 uncalibrated images*” by Y. Wang et al. presents a method to compute projective invariants of 3D points from four uncalibrated images directly.

In the paper entitled “*Comparative study of multimodal biometric recognition by fusion of iris and fingerprint”* by H. Benaliouche and M. Touahria a novel combination of iris and fingerprint biometrics is presented in order to achieve best compromise between a zero FAR and its corresponding FRR.

In the paper “*Design of jitter compensation algorithm for robot vision based on optical flow and Kalman filter*” by B. R. Wang et al. the condition number of coefficient matrix was proposed to quantificationally analyse the effect of matching errors on parameters solving errors.

The paper entitled* “Dynamic multiobjective optimization algorithm based on average distance linear prediction model*” by Z. Li et al. defines a kind of dynamic multiobjective problem with translational Pareto-optimal set (DMOP-TPS) and proposes a new prediction model named ADLM for solving DMOP-TPS.

The aim of the paper entitled “*Application of genetic algorithm to hexagon-based motion estimation”* by C.-M. Kung et al. is to propose a new technique which focuses on combing the hexagon-based search algorithm, which is faster than diamond search, and genetic algorithm.

The paper entitled “*Dynamic scene stitching driven by visual cognition model*” by L.-h. Zou et al. investigates dynamic video sequence stitching, especially under the situation that the scene, captured on a movable platform, contains moving objects or other important interesting regions.

Q. Zhang et al. in the paper entitled “*Automatic recognition of seismic intensity based on RS and GIS: a case study in Wenchuan Ms8.0 earthquake of China*” propose a RS/GIS-based approach for automatic recognition of seismic intensity, in which RS is used to retrieve and extract the information on damages caused by earthquake, and GIS is applied to manage and display the data of seismic intensity.

In the paper entitled “*A robust H.264/AVC video watermarking scheme with drift compensation*” by X. Jiang et al. a robust video watermarking scheme with drift compression is proposed. The devised MB selection scheme can lower the influence on video quality and reduce the possibility of drift distortion.

A novel efficient statistical feature selection approach called improved feature selection based on effective range (IFSER) is proposed in the paper entitled “*An improved feature selection based on effective range for classification*” by J. Wang et al.

In the paper entitled “*Modeling of task planning for multirobot system using reputation mechanism*” by Z. Shi et al. a task planning method based on reputation mechanism is proposed. Reputation plays an important role in the collaboration among people.

The paper entitled “*P-bRS: a Physarum-based routing scheme for wireless sensor networks*” by M. Zhang et al. proposes a novel* Physarum*-based routing scheme (P-bRS) for WSNs to balance routing efficiency and energy equilibrium.

The paper entitled “*Reliability prediction of ontology-based service compositions using petri net and time series models*” by J. Li et al. presents a comprehensive dependability prediction model for OWL-S processes.

A novel approach described to aid breast cancer detection and classification using digital mammograms is presented by X.-S. Zhang in his paper entitled “*A new approach for clustered MCs classification with sparse features learning and TWSVM.*” The proposed method is based on sparse feature learning and representation, which expresses a testing sample as a linear combination of the built vocabulary (training samples).

The paper entitled “*The study of cooperative obstacle avoidance method for MWSN based on flocking control*” by Z. Chen et al. studied the features of target tracking in mobile wireless sensor network and the concept, features, category, application areas of flocking control mode, and obstacle avoidance algorithm.

The paper entitled “*A systematic comparison of data selection criteria for SMT domain adaptation*” by L. Wang et al. performs an in-depth analysis of three different sentence selection techniques.

The paper entitled “*An exponentiation method for XML element retrieval*” by T. Wichaiwong investigates retrieval techniques and related issues over a strongly structured collection using the exponentiation weight for the document's structure over the content-and-structure query, in the data-centric track of the INEX 2011.

In the paper “*Research and application for grey relational analysis in multigranularity based on normality grey number*” by J. Dai et al., combining with the probability distribution of the data, the conception of the normality grey number is proposed.

The paper entitled “*Trusted computing strengthens cloud authentication*” by E. Ghazizadeh et al. proposes the use of trusted computing, federated identity management, and OpenID Web SSO to solve identity theft in the cloud.

The paper by M.-D. Yang et al. entitled “*An efficient fitness function in genetic algorithm classifier for landuse recognition on satellite images*” proposes a new index, DBFCMI, by integrating two common indices, DBI and FCMI, in a GA classifier to improve the accuracy and robustness of classification.

The paper entitled “*Unregistered biological words recognition by Q-learning with transfer learning*” by F. Zhu et al. proposes a novel approach to recognize words based on transfer learning, by which we turn the process of recognizing the terms into a property marking process by redefining the property of terms according to features of terms and the corresponding context.

The paper “*Measuring semantic relatedness between Flickr images: from a social tag based view*” by Z. Xu et al. mainly discusses the semantic relatedness measures systematically, puts forward a method to measure the semantic relatedness of two images based on their tags, and justifies its validity through the experiments.

In the paper entitled “*A GA-based approach to hide sensitive high utility itemsets*” by C.-W. Lin et al., a GA-based algorithm is proposed to find the feasible combination for data sanitization. Each gene in a chromosome represents a possible transaction to be inserted.

The paper entitled “*A novel macroblock level rate control method for stereo video coding*” by G. Zhu et al. proposes a novel macroblock (MB) level rate control method based on binocular perception.

The paper entitled “*Efficient parallel video processing techniques on GPU: from framework to implementation*” by H. Su et al. proposes serial optimization methods, including the multiresolution multiwindow for motion estimation, multilevel parallel strategy to enhance the parallelism of intracoding as much as possible, component-based parallel CAVLC, and direction-priority deblocking filter.

The paper “*Genetic algorithm and graph theory based matrix factorization method for online friend recommendation*” by Q. Li et al. proposes a hybrid genetic algorithm and graph theory based online recommendation algorithm. Hybrid genetic algorithm is used for multiobjective combinatorial problem.

The paper entitled “*Dynamic cooperative clustering based power assignment: network capacity and lifetime efficient topology control in cooperative ad hoc networks*” by X.-H. Li et al. proposes a dynamic cooperative clustering based power assignment (DCCPA) algorithm to solve a new topology control problem: network capacity and energy efficient in cooperative wireless ad hoc networks.

In the paper entitled “*A generalized quantum-inspired decision making model for intelligent agent*” by Y. Hu and C. K. Loo, a generalized quantum-inspired decision making model (QDM) is proposed. QDM helps to extend previous research findings and model more complicated decision space.

The paper entitled “*Unsupervised chunking based on graph propagation from bilingual corpus*” by L. Zhu et al. presents a novel approach for unsupervised shallow parsing model trained on the unannotated Chinese text of parallel Chinese-English corpus.

In the paper entitled “*Research on universal combinatorial coding*” by J. Lu et al., the concept of universal combinatorial coding is advanced and the related properties are analyzed. Universal combinatorial coding can stride over the three branches of coding theory.

L. Zhu et al. in their paper entitled “*Unsupervised Chunking based on graph propagation from bilingual corpus*” present a novel approach for unsupervised shallow parsing model trained on the unannotated Chinese text of parallel Chinese-English corpus.

The paper entitled “*Measurement and analysis of P2P IPTV program resource*” by W. Wang et al. focuses on characteristic analysis of program resources, including the distributions of length of program names, the entropy of the character types, and hierarchy depth of programs.

The paper entitled “*Simple-random-sampling-based multiclass text classification algorithm*” by W. Liu et al. investigates the power law distribution and proposes a SRSMTC algorithm for the MTC problem. This algorithm is an excellent industrial algorithm for its easy implementation and convenient transfer to many applications.

In the paper entitled “*An improved artificial bee colony algorithm based on balance-evolution strategy for unmanned combat aerial vehicle path planning*” by B. Li et al. a novel artificial bee colony (ABC) algorithm improved by a balance-evolution strategy (BES) is applied in this optimization scheme.

In the paper entitled “*New similarity of triangular fuzzy number and its application*” by X. Zhang et al., a new method shape's indifferent area and midpoint (SIAM) to measure the similarity of two triangular fuzzy numbers is proposed, which considers the shapes and midpoints of them.

The paper entitled “*Modeling and computing of stock index forecasting based on neural network and Markov chain*” by Y. Dai et al. presents a new forecast method by the combination of improved back-propagation (BP) neural network and Markov chain, as well as its modeling and computing technology.

The paper entitled “*The theoretical limits of watermark spread spectrum sequence*” by N. Jiang and J. Wang proposes LAC&TCC properties and gives the theoretical limits of SS watermarking sequences under the attacks of cropping and translation.

The paper entitled “*A survey on investigating the need for intelligent power-aware load balanced routing protocols for handling critical links in MANETs*” by B. Sivakumar et al. discusses a short survey on the specialized algorithms and protocols related to energy efficient load balancing for critical link detection in the recent literature.

The paper entitled “*Stock price change rate prediction by utilizing social network activities*” by S. Deng et al. proposed a model to generate heuristically optimized trading rules by utilizing social network activities and historical traded prices and transaction volumes. The proposed model extracts three kinds of features from multiple sources.

The paper entitled “*Ontology-based multiple choice question generation*” by M. Al-Yahya aims to address this issue by assessing the performance of these systems in terms of the efficacy of the generated MCQs and their pedagogical value.

The paper entitled “*A simulation approach to decision making in IT service strategy*” by E. Orta and M. Ruiz explores the use of simulation modeling in this scope and the works founded show that different simulation approaches have been used.

The paper entitled “*A hybrid approach to protect palmprint templates*” by H. Liu et al. proposes a hybrid approach that combines random projection and fuzzy vault to improve the performances at these three points.

The paper entitled “*OntoTrader: an ontological web trading agent approach for environmental information retrieval*” by L. Iribarne et al. showed how traditional traders, properly extended to operate in WIS, are a good solution for information retrieval.

The paper entitled “*An effective news recommendation method for microblog user*” by W. Gu et al. proposed NEMAH system architecture to tackle the personalized news recommendation based on microblog and subclass popularity prediction.

The paper entitled “*ReHypar: a recursive hybrid chunk partitioning method using NAND-flash memory SSD*” by J. No et al. presents a new form of hybrid data allocation scheme, called recursive hybrid chunk partitioning (ReHypar), which can be used in the hybrid structure whose address space is organized by integrating a small portion of NAND-flash SSD partition with the much larger HDD partition.

In the paper entitled “*BgCut: automatic ship detection from UAV images*” by C. Xu et al., an improved universal background model based on Grabcut algorithm is proposed to segment foreground objects from sea automatically.

The paper entitled “*Chinese unknown word recognition for PCFG-LA parsing*” by Q. Huang et al. investigates the recognition of unknown words in Chinese parsing. Two methods are proposed to handle this problem.

I. Fister Jr. et al. in the paper entitled “*A novel hybrid self-adaptive bat algorithm*” have hybridized this algorithm using different DE strategies and applied these as a local search heuristics for improving the current best solution directing the swarm of a solution towards the better regions within a search space.

L. Yao and M. Ling in the paper entitled “*An improved mixture-of-Gaussians background model with frame difference and blob tracking in video stream*” adopt a blob tracking method to cope with this situation.

G. Song and Y. Ye in their paper entitled “*A dynamic ensemble framework for mining textual streams with class imbalance*” propose a new ensemble framework, clustering forest, for learning from the textual imbalanced stream with concept drift (CFIM).

In the paper entitled “*Research on dynamic routing mechanisms in wireless sensor networks*” by A. Q. Zhao et al., a collection tree protocol based, dynamic routing mechanism was proposed for WirelessHART network.

M. Dabbagh and S. P. Lee in the paper entitled “*An approach for integrating the prioritization of functional and nonfunctional requirements*” propose an approach which aims to integrate the process of prioritizing functional and nonfunctional requirements.

Y. Fu et al. in the paper entitled “*A collaborative recommend algorithm based on bipartite community*” propose a bipartite community partitioning algorithm according to the real data environment of collaborative recommendation.

In the paper entitled “*A novel two-stage illumination estimation framework for expression recognition*” by Z. Zhang et al., a two-stage illumination estimation framework is proposed based on three-dimensional representative face and clustering, which can estimate illumination directions under a series of poses.

A. Jimeno-Morenilla et al. in their paper entitled “*Trajectory-based morphological operators: a model for efficient image processing*” propose a new model for computing mathematical morphology operations, the so-called morphological trajectory model (MTM), in which a morphological filter will be divided into a sequence of basic operations.

M. B. Abello and Z. Michalewicz in the paper entitled* “Multiobjective resource-constrained project scheduling with a time-varying number of tasks*” propose an innovative evolutionary algorithm-based approach, called mapping of task ID for centroid-based adaptation with random immigrants (McBAR) and applied this to search for optimal schedules as solutions to the problems.

K. Rujirakul et al. in the paper entitled “*PEM-PCA: a parallel expectation-maximization PCA face recognition architecture*” present the parallel architecture proposal including the first PCA optimization (EM-PCA) and the second parallel face recognition architecture in three substages (PEM-PCA).

In the paper “*iSentenizer-*μ*: Multilingual Sentence Boundary Detection Model*” D. F. Wong et al. present a multilingual sentence boundary detection system (iSentenizer-*μ*) for Danish, German, English, Spanish, Dutch, French, Italian, Portuguese, Greek, Finnish, and Swedish languages. The proposed system is able to detect the sentence boundaries of a mixture of different text genres and languages with high accuracy.

H. Liu et al. in the paper entitled “*Palmprint based multidimensional fuzzy vault scheme*” present a multidimensional fuzzy vault scheme (MDFVS) in which a general subspace error-tolerant mechanism is designed and embedded into FVS to handle intraclass variances.

L. Tian et al. in the paper entitled “*A relationship: word alignment, phrase table, and translation quality*” focus on formulating such a relationship for estimating the size of extracted phrase pairs given one or more word alignment points.

Y. Hu et al. in the paper entitled “*The application of similar image retrieval in electronic commerce*” focus on the online marketing platform based on similar image retrieval that connects information platform with purchase platform.

In the paper entitled “*Towards emotion detection in educational scenarios from facial expressions and body movements through multimodal approaches*,” by M. Saneiro et al., an annotation methodology to tag facial expression and body movements that conform to changes in the affective states of learners while dealing with cognitive tasks in a learning process is presented.

The paper entitled “*A novel resource management method of providing operating system as a service for mobile transparent computing*” by Y. Xiong et al. presents a framework for mobile transparent computing. It extends the PC transparent computing to mobile terminals.

In the paper entitled “*Distributed SLAM using improved particle filter for mobile robot localization*” by F. Pei et al., the improved particle filter was proposed to estimate the state vector of distributed SLAM. The performance of the particle filter is affected by several factors in its operation.

A. L.-F. Han et al. in their paper entitled “*Unsupervised quality estimation model for English to German translation and its application in extensive supervised evaluation*” propose an unsupervised MT evaluation metric using universal part-of-speech tagset without relying on reference translations.

The paper entitled “*Characterizing the effects of intermittent faults on a processor for dependability enhancement strategy*” by C. (Saul) Wang et al. focuses on the effects of intermittent faults on NET versus REG on one hand and the implications for dependability strategy on the other.

The aim of the paper entitled “*A system for sentiment analysis of colloquial Arabic using human computation*” by A. S. Al-Subaihin and H. S. Al-Khalifa is to design and implement a system that can extract the sentiments of Arabic content found in Arabic websites that has informal nature.

The paper entitled “*Moving object detection using dynamic motion modelling from UAV aerial images*” by A. F. M. S. Saif et al. presents moments based motion parameter estimation called dynamic motion model (DMM) to limit the scope of segmentation called SUED which influences overall detection performance.

B. Li et al. in their paper entitled “*A novel artificial bee colony algorithm based on internal-feedback strategy for image template matching*” choose the well-known normalized cross correlation model as a similarity criterion. The searching procedure for the best-match location is carried out through an internal-feedback artificial bee colony (IF-ABC) algorithm.

In the paper entitled “*Development of biological movement recognition by interaction between active basis model and fuzzy optical flow division*” by B. Yousefi and C. K. Loo, a human action recognition method has been proposed; this method is based on interrelevant calculated motion and form information followed the biologically inspired system.

In the paper entitled “*Algorithm for image retrieval based on edge gradient orientation statistical code*” by J. Zeng et al., the statistical method of 8-direction chain code was applied to the statistics for edge gradient direction, to propose a novel edge gradient orientation statistical code (EGOSC), which was sequentially used as feature vector to represent the shape.

In the paper entitled “*A new gravitational particle swarm optimization algorithm for the solution of economic emission dispatch in wind-thermal power system*” by S. Jiang et al., a new hybrid optimization approach, namely, gravitational particle swarm optimization algorithm (GPSOA), is proposed in this paper to solve economic emission dispatch (EED) problem including wind power.

In the paper entitled “*An efficient hierarchical video coding scheme combining visual perception characteristics”* by P. Liu and K. Jia, a hierarchical video coding scheme based on human visual systems (HVS) is proposed in this paper. The proposed scheme uses a joint video coding framework that consists of visual perception analysis layer (VPAL) and video coding layer (VCL).

K.-K. Tseng et al. in the paper entitled “*Signal waveform detection with statistical automaton for internet and web service streaming*” propose an approach to signal waveform detection for Internet and Web streaming, with novel statistical automatons.

K. Hournkumnuard et al. in the paper entitled “*Parallel simulation of HGMS of weakly magnetic nanoparticles in irrotational flow of inviscid fluid*” present the process of high gradient magnetic separation (HGMS) using microferromagnetic wire for capturing the weakly magnetic nanoparticles in the irrotational flow of inviscid fluid.

The paper entitled “*Creation of reliable relevance judgments in information retrieval systems evaluation experimentation through crowdsourcing: a review*” by P. Samimi and S. D. Ravana is intended to explore different factors that have an influence on the accuracy of relevance judgments accomplished by workers and how to intensify the reliability of judgments in crowdsourcing experiment.

In the paper entitled “*Monte Carlo method with heuristic adjustment for irregularly shaped food product volume measurement*” by J. Siswantoro et al., a nondestructive method for volume measurement of irregularly shaped food products that is based on the Monte Carlo method using a computer vision system is proposed.

